# TIPE2 May Target the Nrf2/HO-1 Pathway to Inhibit M1 Macrophage–Related Neutrophilic Inflammation in Asthma

**DOI:** 10.3389/fimmu.2022.883885

**Published:** 2022-04-28

**Authors:** Bingqing Shi, Yuqiu Hao, Wei Li, Hongna Dong, Mengting Xu, Peng Gao

**Affiliations:** Department of Respiratory Medicine, The Second Hospital of Jilin University, Changchun, China

**Keywords:** asthma, phenotype, inflammation, macrophages, TIPE2

## Abstract

**Purpose:**

Although recent studies have highlighted the link of TIPE2 and asthma airway inflammation, its roles and molecular mechanisms in different asthma inflammatory phenotypes remain largely unknown. We evaluated sputum TIPE2 expression level and its correlation with different asthma phenotypes. Additionally, we explored the roles and mechanism of TIPE2 in M1 polarization of macrophages.

**Methods:**

A total of 102 asthma patients who underwent sputum induction were enrolled to evaluate the expression level of TIPE2 and its association with different asthma phenotypes. To explore the roles and mechanism of TIPE2 in M1 polarization of macrophages, THP-1 monocytes stimulated with phorbol-12-myristate-13-acetate, were used as a model of undifferentiated (M0) macrophages, and M0 macrophages were treated with lipopolysaccharide to induce M1 macrophages.

**Results:**

The sputum TIPE2 level was significantly lower in patients with neutrophilic asthma (NA) and higher in patients with eosinophilic asthma (EA) compared with patients with paucigranulocytic asthma. The levels of IL-1β, TNF-α and IL-6 were highest in NA compared with other groups. TIPE2 levels in sputum negatively correlated with IL-1β and TNF-α levels but positively correlated with IL-4, IL-5, IL-13, and IL-10 levels (*P* < 0.05). *In vitro*, TIPE2 enhanced Nrf2/HO-1 pathway activation in macrophages and inhibited LPS-induced M1 macrophage differentiation and related cytokine release. Further analysis showed that the Nrf2 inhibitor ML385 weakened TIPE2-induced activation of the Nrf2/HO-1 pathway, as well as TIPE2-induced suppression in M1 polarization of macrophage and inflammatory cytokines secretion.

**Conclusions:**

TIPE2 expression level was highly down-regulated in NA and was negatively correlated with inflammatory factors (IL-1β and TNF-α). Aberrant expression of TIPE2 may target the Nrf2/HO-1 pathway to inhibit M1 macrophage–related neutrophilic inflammation in asthma.

## Introduction

Asthma is a complex and heterogeneous disease characterized by chronic airway inflammation, airway hyperresponsiveness (AHR), and reversible airflow limitation (1). The inflammatory response in asthma is driven by recruitment of the Th2/Th1 lymphocytes, neutrophils, eosinophils, and macrophages to the lung and is associated with M1/M2 polarization of macrophages (2, 3). Asthma is divided into four major inflammatory phenotypes based on the percentages of eosinophils and neutrophils in induced sputum of patients: eosinophilic asthma (EA), neutrophilic asthma (NA), mixed granulocytic asthma (MA), and paucigranulocytic asthma (PA) (4–6). Eosinophilic inflammation is the most common type of asthmatic airway inflammation (7). The main molecular mechanism of airway inflammation in EA is type 2 inflammation, which is driven by T helper (Th) 2 responses and mediated by interleukin (IL)-4, IL-5, and IL-13 (8). Corticosteroids and biological therapies (e.g., anti-IL-4Rα, anti-IL-5/5Rα, and anti-IgE antibodies) have been indicated for asthma treatment in the guidelines of the Global Initiative for Asthma (GINA) (9). However, in contrast to EA patients, some patients with NA exhibit neutrophil dominance airway inflammation that can be driven by M1 macrophages or Th1/Th17 lymphocytes (3, 10, 11). Thus, patients with NA respond poorly to corticosteroids and biological therapies (12, 13) and commonly progress to severe asthma or refractory asthma (14). Therefore, further research on the possible mechanisms and biomarkers for non-EA phenotypes is necessary to identify effective treatment strategies for these patients.

Tumor necrosis factor-α-induced protein 8 (TNFAIP8, also known as TIPE)–like 2 (TIPE2), a member of the TNFAIP8 family, maintains immune homeostasis and regulates inflammation (15–17). TIPE2 gene deletion in mice led to the spontaneous development of lethal inflammation of multiple organs, including the lung (16). TIPE2 regulates macrophage polarization and Th cell–mediated immune responses *in vivo*, as well as neutrophil and eosinophil activity and exudation (18–20). TIPE2 promoted the immune-suppressive effects of Tregs by increasing Foxp3 expression, thereby extenuating airway inflammation and airway hyperresponsiveness in the asthma mouse model (21). One study showed that TIPE2 levels in peripheral blood mononuclear cells (PBMCs) of asthma patients were lower than levels in healthy individuals and negatively correlated with eosinophil, IL-4, and IgE levels (22). However, another report found that TIPE2 expression in the polyps of asthma patients with eosinophilic chronic rhinosinusitis with nasal polyps (Eos CRSwNP) was significantly increased compared with expression in patients with non-asthmatic Eos CRSwNP and was positively correlated with eosinophils and local eosinophilic inflammation (23). Therefore, TIPE2 may be related to airway inflammation in asthma. However, the role of TIPE2 in asthma is complex and remains unclear.

Nuclear factor E2-related factor 2 (Nrf2) is an important transcription factor that promotes the gene expression of cytoprotective gene heme oxygenase-1 (HO-1), thereby reducing the damage caused by oxidative stress and inflammation in the body (24). HO-1 is an antioxidant enzyme expressed by macrophages (25). Induced expression of HO-1 significantly inhibited lipopolysaccharide (LPS)-induced M1 macrophage polarization and related proinflammatory cytokine gene expression (26). In addition, activation of the Nrf2 pathway significantly reduced Th1 and Th17 cytokine expression (27) and attenuated LPS-induced neutrophil recruitment (28). Whether TIPE2 exerts anti-inflammation activities *via* Nrf2/HO-1 is unclear.

The heterogeneity of asthmatic airway inflammation and the variability of asthma phenotypes may contribute to the disparate roles of TIPE2 in asthma. Here, we sought to determine the expression pattern of TIPE2 in different asthma phenotypes and its relationship with neutrophilic and eosinophilic inflammation. In addition, we further investigated the possible cellular and molecular signaling of TIPE2 in NA. We speculate that TIPE2 may mediate the M1 polarization of macrophages by activating the Nrf2/HO-1 pathway to alleviate airway neutrophilic inflammation in asthma.

## Methods

### Study Subjects

This cross-sectional study included 113 patients with asthma recruited from the Department of Respiratory Medicine of Jilin University Second Hospital. Asthma was diagnosed in accordance with the 2012 GINA criteria (29). Subjects underwent fractional exhaled nitric oxide (FeNO) tests, pulmonary function tests, and sputum induction. The Asthma Quality of Life Questionnaire (AQLQ) was used to assess patient quality of life, and the 6-item Asthma Control Questionnaire (ACQ6) and Asthma Control Test Questionnaire (ACT) were used to assess asthma control. All examinations were conducted on the same day. Ethical approval was received from the Ethics Committee of the Second Hospital of Jilin University (approval number: 2016-34).

### Sputum Induction

Sputum induction was carried out according to previously described procedures (30, 31). Briefly, participants underwent 15 min of sputum induction with 4.5% hypertonic saline. Dithiothreitol was used to disperse the sputum cells, and the cells were resuspended in 1× phosphate buffered saline, and the total cell count was determined. Cytospins were prepared and stained by May–Grunwald– Giemsa. Cell counts of inflammatory cells (eosinophils, neutrophils, macrophages, epithelial cells, and lymphocytes) were calculated.

### Sputum Inflammatory Phenotype Classification

According to previously published criteria, the granulocyte cutoff values were 3% for sputum eosinophils (5) and 61% for sputum neutrophils (6). The EA phenotype was defined as sputum eosinophil counts ≥3%; the NA phenotype was defined as neutrophil counts ≥61%; the MA was defined as eosinophil counts ≥3% and neutrophil counts ≥61%; and the PA phenotype was defined as eosinophil counts < 3% and neutrophil counts < 61%.

### Measurement of TIPE2 and Other Cytokines

Quantitative detection of sputum TIPE2 and sputum IL-1β was performed using the commercial human TNFAIP8L2 ELISA Kit (Catalog Number EK6248-2, Signalway Antibody, USA) and Human IL-1β ELISA Kit (CSB-E08053h, CUSABIO, Wuhan, China) according to the manufacturers’ protocols. The levels of other cytokines (IL-2, IL-4, IL-5, IL-6, IL-9, IL-10, IL-13, IL-17A, IL-17F, IL-22, IL-23, TNF-α, and interferon (IFN)-γ) were measured using the Cytometric Bead Array under the Multi-Analyte Flow Assay Kit (Biolegend, USA) following the manufacturer’s instructions. All incubation steps were performed at room temperature and protected from light. LEGENDplex v8.0 software was used to analyze the data.

### Cell Culture

The human monocyte leukemia cell line (THP-1) was obtained from the Cell Bank of the Type Culture Collection of the Chinese Academy of Sciences. The cells were cultured in RPMI Medium 1640 basic (1X) supplemented with 10% FBS and 1% penicillin/streptomycin solution (Thermo Fisher Scientific, Inc., USA) under standard conditions (37°C and 5% CO_2_ in a humidified incubator). THP-1 cells were cultured with phorbol-12-myristate-13-acetate (PMA, 20 ng/ml; Cat. #M4647, AbMole BioScience) for 48 h to transform into undifferentiated (M0) macrophages. After culture without PMA for 24 h, the M0 macrophages were treated with lipopolysaccharide (LPS, 1 µg/ml; Cat. #L2880, Sigma-Aldrich) for 24 h to induce M1 macrophages.

### Cell Transfection

To overexpress or silence the TIPE2 gene in THP-1, cells were transduced by recombinant lentivirus carrying a TIPE2 over-expression vector (TIPE2-OE) or small hairpin RNA (shRNA) targeting the TIPE2 gene (sh-TIPE2) for 6 h, followed by incubation under standard growth conditions. At 72 h after transfection, overexpression or depletion of TIPE2 was confirmed by qRT-PCR and western blotting.

The production and packaging of the human TIPE2 overexpression vector and the viral vector was conducted by Beijing Mijia Biotech Company. The human TIPE2 sequence was derived from the NCBI database (NM_024575.5) and the TIPE2 shRNA sequence was CCATGACGGCACTTAGCTTTG.

### Nrf2 Inhibitor

ML385 (32), a small molecule inhibitor of Nrf2, was purchased from TargetMol Company (Catalog Number: T4360, Shanghai, China).

### Immunofluorescence Analysis

TIPE2 immunofluorescent staining was performed on sputum cytospin smears from asthma patients. Expression of Nrf2 in LPS-induced M1 macrophages was examined by immunofluorescence following treatment with sh-TIPE2, TIPE2-OE or ML385. Briefly, after pretreatment, sputum single cell smears or cell slides were fixed for 30 min or overnight in 4% paraformaldehyde, followed by incubation with primary antibody overnight at 4°C. Cells were then incubated with CY3-conjugated goat Anti-Rabbit IgG (H+L) (E-IR-R321, Elabscience, Wuhan, China) for 60 min at 37°C in the dark. Nuclei were stained with DAPI. Samples were then analyzed using a fluorescence microscope (Olympus, Japan). The primary antibodies included antibodies against TIPE2 (catalog number: 15940-1-AP, 1:100, Proteintech, Wuhan, China) and Nrf2 (Cat. #ab62352, 1:500, Abcam).

### Real-Time Quantitative PCR (RT-qPCR)

Total RNA was isolated using the Cell Total RNA Isolation Kit (FOREGENE, Chengdu, China) according to the manufacturer’s protocols, and RNA was reverse transcribed using the StarScript II First-strand cDNA Synthesis Mix (GenStar, Beijing, China). RT-qPCR was carried out with the CFX96 Touch™ Real-Time qPCR Detection System (BIO-RAD, USA). Results were analyzed using the 2^−ΔΔCt^ method. GAPDH mRNA was used as an internal control. The primer sequences are provided in **Table 1**.

**Table 1:** Primer sequences for real time PCR.

Genes	Sense primers	Antisense primers
GAPDH	GTCTCCTCTGACTTCAACAGCG	ACCACCCTGTTGCTGTAGCCAA
TNFAIP8L2	CTGAGTAAGATGGCGGGTCG	TGCGTGTACTCCTTGGACAC
IL-1β	CCACAGACCTTCCAGGAGAATG	GAGCAGTTCAGTGATCGTACAGG
IL-6	AGACAGCCACTCACCTCTTCAG	TTCTGCCAGTGCCTCTTTGCTG
TNF-α	CAGCCTCTTCTCCTTCCTGAT	GCCAGAGGGCTGATTAGAGA
CD11c	GATGCTCAGAGATACTTCACGGC	CCACACCATCACTTCTGCGTTC
iNOS	GACATGGTGCAGAGCAACC	CAGCACCAGTGTGTTGGTTC
Nrf2	CGGTATGCAACAGGACATTG	TCTGTCAGTTTGGCTTCTGG
HO-1	CCAGGCAGAGAATGCTGAGTTC	AAGACTGGGCTCTCCTTGTTGC

### Western Blot Analysis

Western blot was performed as previously described (33). Primary antibodies against Nrf2 (cat. #ab62352) and HO-1 (cat. #ab13248) were purchased from Abcam (Cambridge, MA, USA) and used at a dilution 1:1000. The anti-Nrf2 antibody used in our study targets Nrf2 protein with a molecular weight of ~100 kDa. Antibodies against β-actin (1:5000 dilution, Cat. #66009-1-lg, Proteintech, San Ying Biotechnology, China), Tubulin (1:20000, Cat. # 66031-1-Ig, Proteintech) and Lamin B1 (1:2000, Cat. #12987-1-AP, Proteintech) served as internal controls. Appropriate secondary antibodies (Proteintech) were used.

### Subcellular Fractionation

The nuclear and cytoplasmic protein extraction was performed using the nuclear extraction kit from BestBio (Cat. #BB-3115, Shanghai, China) according to manufacturer’s instructions.

### ELISA

Cell culture supernatants were collected for cytokine assay by ELISA (Elabscience, Wuhan, China), performed according to the manufacturer’s instructions. Levels of IL-6, IL-1β and TNF-α were detected.

### Statistical Analysis

IBM SPSS Statistics, version 25.0, was used for statistical analysis. Normally distributed data were expressed as the mean ± standard deviation (SD) and a comparison of two groups was performed using Student’s t-test. Comparison of the means among three or more groups was performed using one-way analysis of variance (ANOVA) followed by *post-hoc* test (least significant difference [LSD] or Tamhane’s T2). Non-normally distributed data were summarized as the median (Q1, Q3) and comparisons between subgroups were performed using Kruskal–Wallis test accompanied by Bonferroni correction or Mann–Whitney U test. For categorical variables, the data were expressed as percentages, and the chi-square test was used for analysis. Spearman correlation coefficients were used to measure the association between clinical parameters of the patients. p<0.05 was considered statistically significant.

## Results

### Demographic and Clinical Characteristics of Asthma Patients and Healthy Volunteers

This study included 113 patients with asthma and 22 healthy volunteers (**Table 2**). No significant differences in patient age, sex, body mass index (BMI), percentage of smokers, and smoking index were detected between asthma patients and healthy volunteers (p > 0.05). Compared with the healthy group, the asthma patients had significantly reduced lung function (FEV1/FVC, FEV1% predicted) (p < 0.001). The sputum eosinophil percentage was higher in asthma patients compared with healthy volunteers and the sputum macrophage percentage was lower (p < 0.05).

**Table 2:** Demographic, functional, and inflammatory characteristics of asthma patients and healthy volunteers.

	Asthma patients	Healthy volunteers	P value
N	113	22	
Age, years	55 ± 15	54 ± 14	0.747
Sex (M/F)	50/63	10/12	0.917
BMI (kg/m^2^)	23.3 ± 3.5	23.7 ± 2.6	0.642
Ex-smokers, n (pack-years)	25 (22.1)	4 (18.2)	0.278
Current smokers, n (%)	21 (18.6)	6 (27.3)	0.828
Smoking index	25 (13.5,40.0)	0 (0,5.6)	<0.001
Pre-FEV1	2.0 ± 0.8	2.7 ± 0.6	<0.001
Pre-FVC	3.3 ± 1.0	3.4 ± 0.7	0.019
FEV1/FVC (%)	61.3 ± 12.4	79.2 ± 6.2	<0.001
FEV1% predicted	67.4 ± 24.4	98.4 ± 16.1	<0.001
Sputum TCC (10^6^/mL)	2.1 (0.6,5.2)	2.4 (1.4,5.2)	0.218
Sputum eosinophils, %	1.8 (0.1,5.2)	0.1 (0,0.4)	<0.001
Sputum neutrophils, %	7.3 (0.6,53.3)	9,6 (4.1,22.1)	0.546
Sputum macrophages, %	60 (29.5,90.0)	84 (72.4,90.6)	0.035
Sputum lymphocytes, %	5.1 (0.9,10.6)	2.0 (1.0,4.2)	0.072
TIPE2 (ng/ml)	0.6 (0.3,1.4)	0.5 (0.3,0.7)	0.806

BMI, body mass index; FEV1, forced expiratory volume in 1 second; FVC, forced vital capacity; TCC, total cell count.

Data are presented as mean ± SD or median (Q1, Q3), unless otherwise indicated.

### Clinical Characteristics and Inflammatory Phenotypes in Asthma

Sputum cell counts from 102 asthma patients were available, and the patients were classified with asthma inflammatory phenotypes as follows: 48 (47.1%) patients had PA, 31 (30.4%) had EA, 12 (11.8%) had NA, and 11 (10.8%) had MA (**Table 3**). There were no significant differences in the average age, sex, BMI, and percentage of smokers among the four groups (p > 0.05). Patients with EA had a significantly higher FeNO value and blood eosinophil count compared with NA and PA groups and a significantly lower blood neutrophil count (p < 0.05).

**Table 3:** Demographic and clinical characteristics according to the inflammatory phenotype.

	NA	EA	MA	PA	P value
N	12	31	11	48	
Sex (M/F)	6/6	13/18	4/7	24/24	0.805
Age (years)	62 ± 8^£^	51 ± 20^þ_^	51 ± 9	52 ± 15	0.077
BMI	23.7 ± 4.2	23.3 ± 5.3	27.6 ± 2.6	23.4 ± 2.8	0.811
Current/ex-smoker, N (%)	7 (58.3)	11 (35.5)	5 (45.5)	23 (47.9)	0.560
Smoking index	39 (9.8,50.0)	17.5 (13.6,35.0)	40 (18.5,45.0)	22 (13.3,40.0)	0.763
Post-FEV1 (L)	2.3 ± 1.1	2.5 ± 0.9	2.5 ± 0.7	2.4 ± 1.0	0.719
Post-FVC (L)	3.6 ± 1.2	3.6 ± 0.9	3.5 ± 0.6	3.7 ± 1.3	0.820
Post-FEV1/FVC, %	60.8 ± 17.6	70.3 ± 15.8	69.4 ± 14.9	66.1 ± 16.5	0.885
FeNO (ppd)	29(18.5,49.7)^£^	59(38.2,91.5)^§þ_^	50(36.8,124.4)^Ψ^	21(13.7,30.5)^‡#^	<0.001
ACQ6	1.1 ± 0.5	1.8 ± 0.9	2.1 ± 2.0	1.5 ± 0.8	0.430
ACT	18.0 ± 4.3	15.2 ± 2.4	18.7 ± 7/7	15.6 ± 3.6	0.688
Global AQLQ	5.3 ± 1.2	4.8 ± 0.9	5.2 ± 1.8	4.8 ± 0.9	0.308
Blood eosinophils, 10^9^/L	0.3 (0.1,0.4)^§^	0.4 (0.2,0.7)^§^	0.2 (0.2,1.6)^§^	0.1 (0.0,0.2)^‡¶†^	<0.001
Blood neutrophils, 10^9^/L	5.7 (4.8,6.7)^‡^	3.4 (2.9,4.3) ^†¶§^	4.9 (4.1,6.0)^‡^	4.7 (3.6,6.0)^‡^	<0.001
Blood lymphocytes, 10^9^/L	1.9 ± 0.3	2.0 ± 0.8	2.5 ± 0.6	1.7 ± 0.5	0.487
Sputum TCC (10^6^/mL)	2.4 (0.5,8.8)	1.8 (1.0,3.3)	3.0 (2.2,5.4)	1.0 (0.7,3.4)	0.170

NA, neutrophilic asthma; EA, eosinophilic asthma; MA, mixed granulocytic asthma; PA, paucigranulocytic asthma; BMI, body mass index; FeNO, fractional exhaled nitric oxide; FEV1, forced expiratory volume in 1 s; FVC, forced vital capacity; ACT, asthma control test; ACQ, asthma control questionnaire; AQLQ, Asthma Quality of Life Questionnaire; TCC, total cell count.

Data are presented as mean ± SD or median (Q1, Q3), unless otherwise indicated.

p<0.01: ^†^vs. NA, ^‡^vs. EA, ^§^vs. PA, ^¶^vs. MA. p<0.05: ^þ_^vs. NA, ^Ψ^vs. PA, ^£^vs. EA, ^#^vs. MA.

### TIPE2 Expression in Asthma Inflammatory Phenotypes

**Figure 1:**
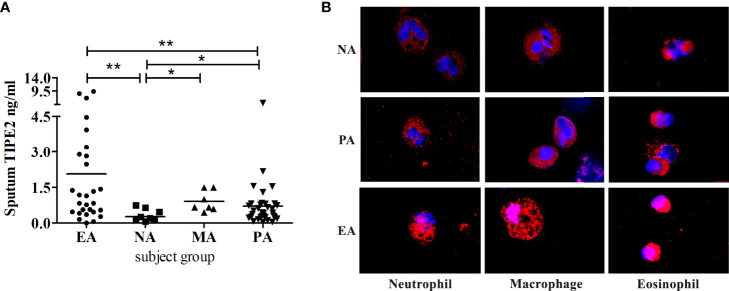
**(A)** Induced sputum concentrations of TIPE2 in the asthma inflammatory phenotypes. **(B)** Sputum cells from NA, PA, and EA patients stained for TIPE2 (red); nuclei were stained with DAPI (blue). NA, neutrophilic asthma; EA, eosinophilic asthma; MA, mixed granulocytic asthma; PA, paucigranulocytic asthma. *P<0.05, **P<0.01.

We detected no significant difference in sputum TIPE2 levels between asthma patients and healthy individuals (p > 0.05) (**Table 2**). However, the sputum TIPE2 level in patients with NA was also significantly lower compared with levels in patients with PA but significantly higher in patients with EA compared with levels in patients with PA (p < 0.05) (**Figure 1A**). We next performed TIPE2 immunofluorescence analysis on sputum inflammatory cells from all asthma phenotypes (**Figure 1B**). TIPE2 expression was lower in sputum neutrophils of all phenotypes compared with sputum eosinophils. Macrophages in NA showed lower levels of TIPE2 staining compared with macrophages in PA, whereas macrophages in EA showed intense expression of TIPE2. All immune cells in NA, including neutrophils, macrophages, and eosinophils, showed lower levels of TIPE2 staining compared with cells in EA (**Figure 1B**). Therefore, sputum TIPE2 expression was down-regulated in neutrophils and NA but was up-regulated in eosinophils and EA. Interestingly, TIPE2 levels in the sputum of patients with MA were significantly higher than those in NA and PA (p < 0.05) but were not significantly different from those in EA (p > 0.05), which may be caused by the different ratio of sputum neutrophils and eosinophils. The expression pattern of TIPE2 in asthma phenotypes may depend on the heterogeneity of airway inflammation.

### Correlation Between Inflammatory Mediators and Clinical Characteristics

**Figure 2:**
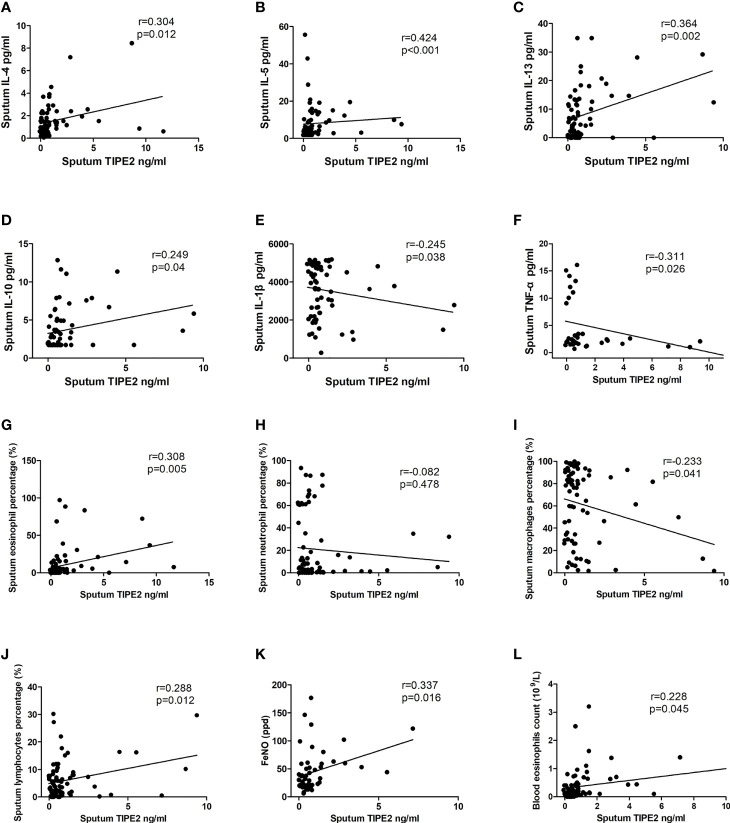
Correlations between TIPE2 and inflammatory mediators/clinical characteristics. TNFAIP8, tumor necrosis factor-alpha-induced protein–8; TIPE, TNFAIP8-like; IL, interleukin; TNF-α, tumor necrosis factor-α; FeNO, fractional exhaled nitric oxide.

Sputum TIPE2 was positively correlated with sputum IL-4, IL-5, IL-13, and IL-10 (p < 0.05) (**Figures 2A–D**). Sputum TIPE2 levels were positively correlated with eosinophils and lymphocytes and negatively correlated with macrophages (p < 0.05) (**Figures 2G, I, J**). Sputum TIPE2 was negatively correlated with sputum IL-1β and TNF-α levels (p < 0.05) (**Figures 2E, F**). There was no significant correlation between sputum TIPE2 and sputum neutrophils (p > 0.05) (**Figure 2H**). Sputum TIPE2 was positively correlated with FeNO value and blood eosinophils count (p < 0.05) (**Figures 2K, L**). In addition, there was no significant correlation between sputum TIPE2 and IL-6 (r=-0.144, p=0.290). We also found that sputum TIPE2 expression was similar in patients taking different doses of inhaled corticosteroid (ICS), and there was no significant correlation between sputum TIPE2 and ICS treatment dose (p > 0.05).

### TIPE2 Impedes M1 Macrophage Differentiation

**Figure 3:**
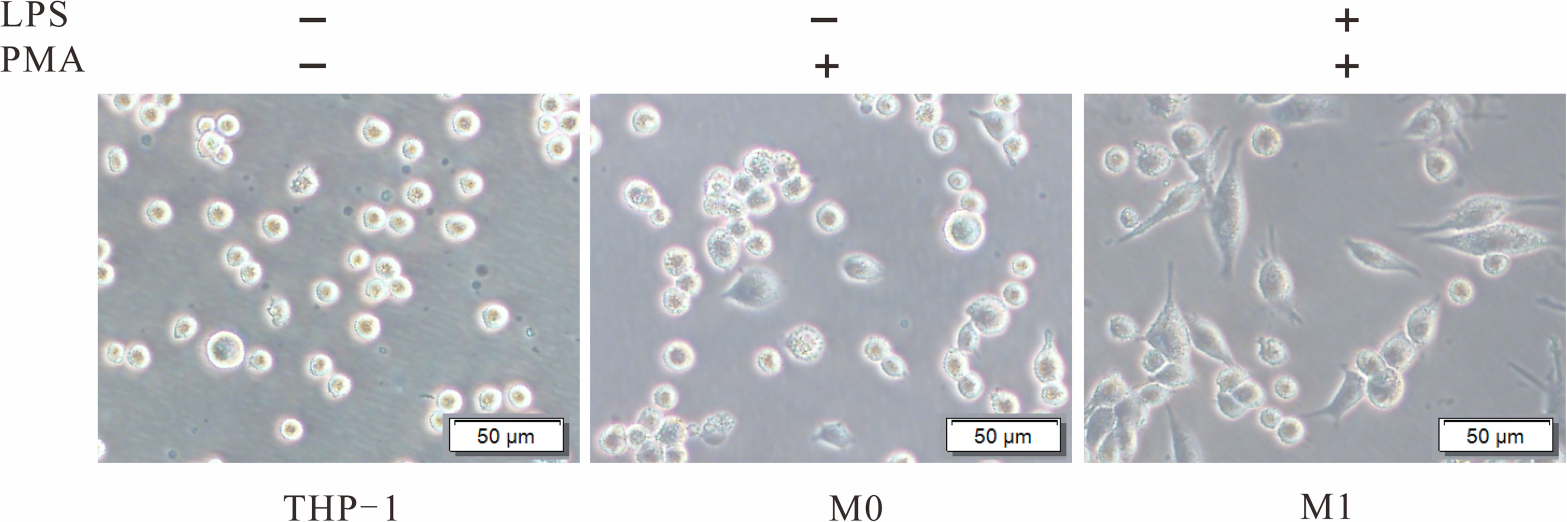
Morphological changes of THP-1 induced by PMA and LPS. THP-1 cells were cultured with PMA (20 ng/ml) for 48 h to transform into M0. After culture without PMA for 24 h, the M0 macrophages were treated with LPS (1 µg/ml) for 24 h to induce M1. PMA, phorbol-12-myristate-13-acetate; LPS, lipopolysaccharide; M0, undifferentiated macrophage; M1, M1 macrophage.

**Figure 4:**
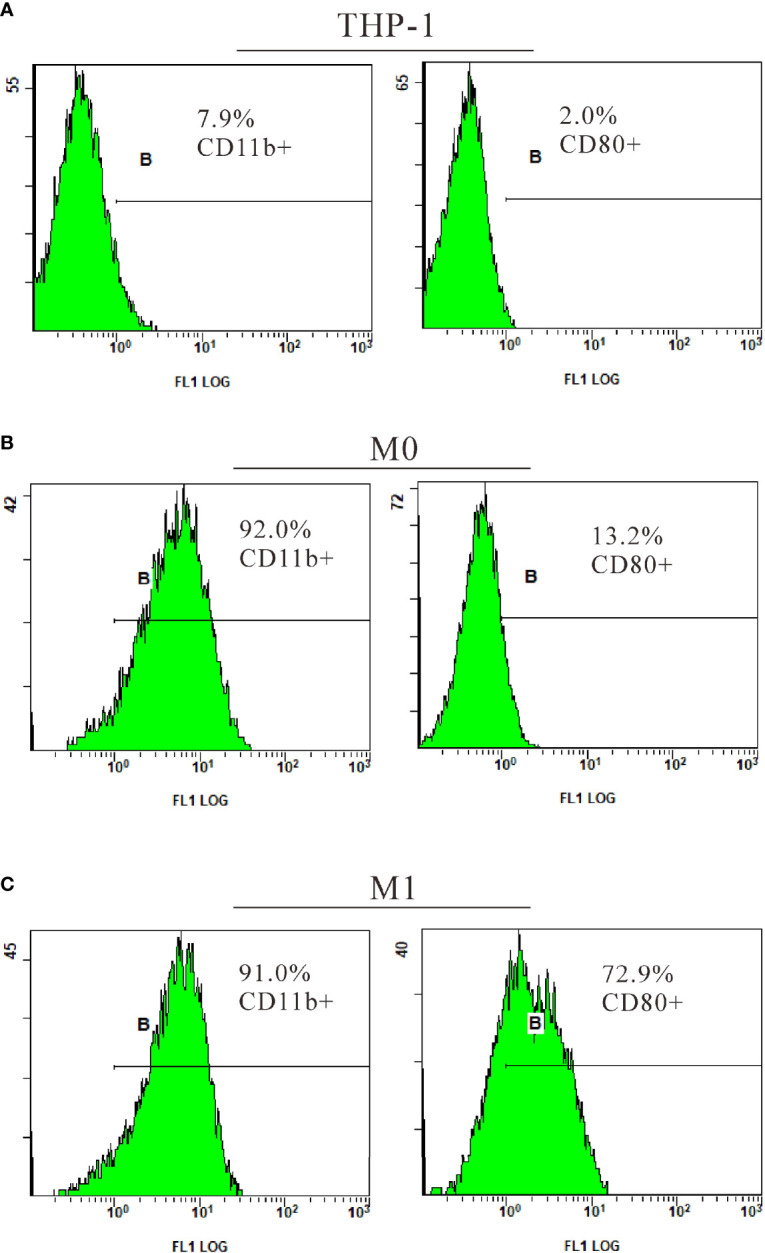
Identification of M1 macrophages by flow cytometry. THP-1 cells were cultured with PMA (20 ng/ml) for 48 h to transform into M0. After culture without PMA for 24 h, the M0 macrophages were treated with LPS (1 µg/ml) for 24 h to induce M1. CD11b, a surface marker of macrophages; CD80, a surface marker of M1 macrophages; PMA, phorbol-12-myristate-13-acetate; LPS, lipopolysaccharide; M0, undifferentiated macrophage; M1, M1 macrophage.

To explore the role of TIPE2 in the M1 polarization of macrophages, THP-1 monocytes stimulated with PMA were used as the model of undifferentiated (M0) macrophages. M0 macrophages were then treated with LPS to induce M1 macrophages. M0 and M1 macrophages were initially identified by morphology under the optical microscope (**Figure 3**). Further analysis by flow cytometry showed a significant increase in CD11b+ cells (7.9% *vs*. 92%) following PMA stimulation of THP-1 (**Figure 4**). After LPS continued to stimulate M0, CD80+ cells increased significantly (13.2% *vs*. 72.9%), while the number of CD11b+ cells exhibited a non-significant change (92% *vs*. 91%) (**Figure 4**). These results support the differentiation of THP-1 cells in terms of M0 and M1 macrophages.

**Figure 5:**
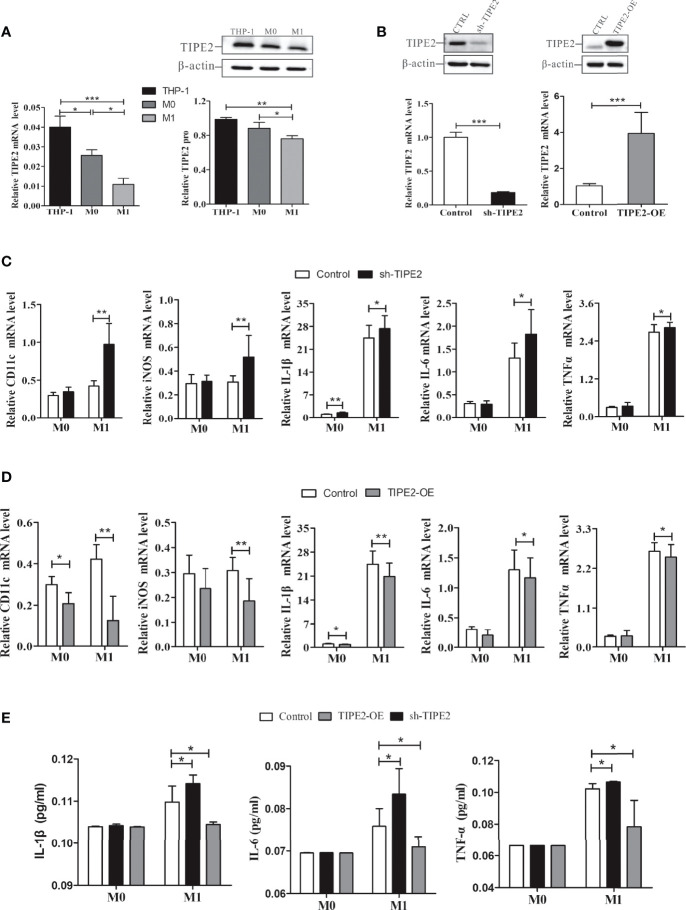
TIPE2 impeded M1 macrophage differentiation and inhibited the expression and release of related inflammatory cytokines. **(A)** TIPE2 expression was down-regulated in M1 macrophages. TIPE2 expression levels were detected by RT-qPCR and western blotting analysis. **(B)** THP-1 monocytes were transduced with lentivirus expressing TIPE2 shRNA or TIPE2 overexpression sequences. TIPE2 expression levels were determined by the same method as above. **(C, D)** Expression of M1 genes in THP-1 monocytes by RT-qPCR following treatment with PMA (20 ng/ml) for 48 h and LPS (1 µg/ml) for 24 h. Measurements of IL-1β, IL-6, and TNF-α mRNA expression levels in THP-1 monocytes by RT-qPCR following treatment with PMA (20 ng/ml) for 48 h and LPS (1 µg/ml) for 2 h. **(E)** Measurement of IL-1β, IL-6, and TNF-α secretion in THP-1 monocytes by ELISA following treatment with LPS (1 µg/ml) for 2 h. Statistics were performed on pooled data from at least three independent experiments. Error bars represent the standard deviations of the means. A comparison of two groups was performed using Student’s t-test. Comparison of the means among three groups was performed using one-way ANOVA followed by *post-hoc* test (LSD or Tamhane’s T2). ^*^P<0.05, ^**^P<0.01, ^***^P<0.001. TIPE2, tumor necrosis factor α–induced protein 8–like protein 2; RT-qPCR, reverse transcription–quantitative polymerase chain reaction; shRNA, short hairpin RNA; TIPE2-OE, TIPE2 overexpression; iNOS, inducible nitric oxide synthase; IL, interleukin; TNF-α, tumor necrosis factor-α; LPS, lipopolysaccharide; PMA, phorbol-12-myristate-13-acetate; CTRL, control vector; ANOVA, analysis of variance; LSD, least significant difference.

M1 macrophages and M1 cytokines (IL-6, IL-1β, and TNF-α) are involved in neutrophilic airway inflammation in asthma (2, 3). Our results showed that TIPE2 mRNA and protein expressions were decreased in M1 macrophages compared with M0 macrophages (**Figures 5A, B**). THP-1 monocytes were then transduced with lentivirus expressing TIPE2 shRNA, and monocytes with TIPE2 shRNA exhibited decreased expression levels of TIPE2 (**Figure 5B**). The expression levels of M1 genes and cytokines in M0 macrophages were measured by RT-qPCR and ELISA following treatment with LPS. Under M1 macrophage–inducing conditions, TIPE2-silenced macrophages exhibited increased expression levels of M1 genes (CD11c and inducible nitric oxide synthase [iNOS] genes) compared with control macrophages (**Figure 5C**). The expressions of IL-1β, IL-6, and TNF-α were also significantly upregulated in TIPE2-silenced M1 macrophages (**Figure 5C**). In addition, compared with control M1 macrophages, M1 macrophages with TIPE2 gene silencing secreted significantly more IL-1β, IL-6, and TNF-α into the supernatant (**Figure 5E**).

Lentivirus was then used to overexpress TIPE2 in macrophages (**Figure 5B**). Compared with the control group, TIPE2 overexpression in macrophages inhibited M1 gene expression and inflammatory cytokine secretion (**Figures 5D, E**). TIPE2 impeded LPS-induced M1 macrophage differentiation and early inflammation.

### TIPE2 Promotes Nrf2/HO-1 Signaling Pathway Activation

**Figure 6:**
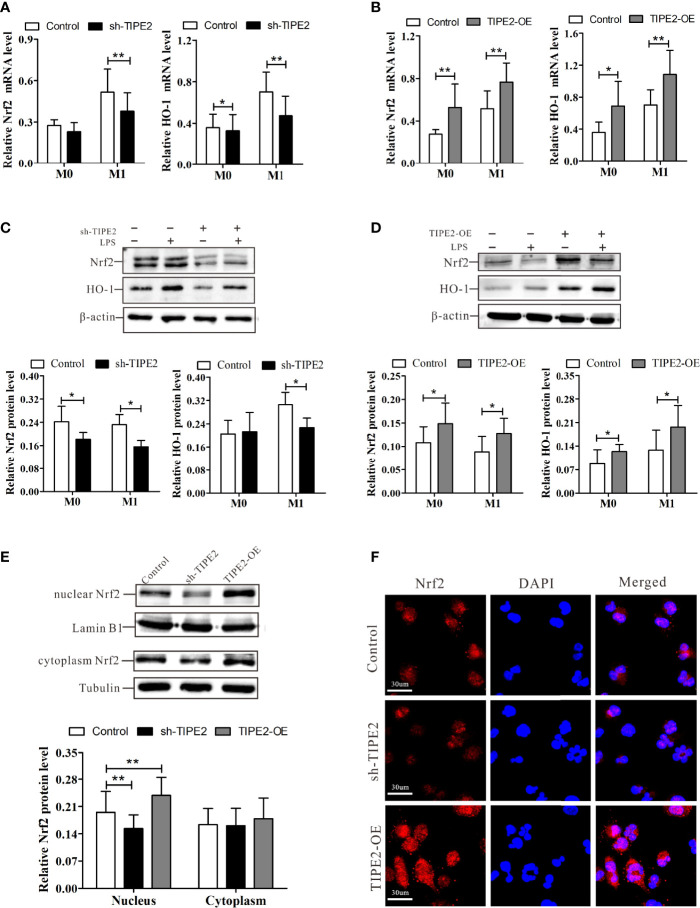
TIPE2 promotes Nrf2/HO-1 signaling pathway activation. **(A, B)** Expression of Nrf2 and HO-1 in THP-1 monocytes by RT-qPCR following treatment with PMA (20 ng/ml) for 48 h and LPS (1 µg/ml) for 24 h. **(C, D)** Nrf2 and HO-1 protein levels in THP-1 monocytes were detected by western blotting analysis following treatment with PMA (20 ng/ml) for 48 h and LPS (1 µg/ml) for 24 h. **(E)** Expression of nuclear and cytoplasmic Nrf2 in M1 macrophages was detected by western blotting analysis. **(F)** Expression of Nrf2 in M1 macrophages was detected by immunocytochemistry analysis. Statistics were performed on pooled data from at least three independent experiments. Error bars represent the standard deviations of the means. A comparison of two groups was performed using Student’s t-test. Comparison of the means among three groups was performed using one-way ANOVA followed by *post-hoc* test (LSD or Tamhane’s T2). ^*^P<0.05, ^**^P<0.01. TIPE2, tumor necrosis factor α–induced protein 8–like protein 2; Nrf2, nuclear factor erythroid 2–related 2; HO-1, heme oxygenase-1; RT-qPCR, reverse transcription–quantitative polymerase chain reaction; shRNA, short hairpin RNA; TIPE2-OE, TIPE2 overexpression; LPS, lipopolysaccharide; PMA, phorbol-12-myristate-13-acetate; CTRL, control vector; ANOVA, analysis of variance; LSD, least significant difference.

Activation of the Nrf2/HO-1 pathway restrains the M1 polarization of macrophages and exerts anti-inflammatory effects (26). We next explored whether TIPE2 regulates Nrf2/HO-1 pathway activation. RT-qPCR analysis demonstrated that macrophages with TIPE2 overexpression showed a significant increase in the mRNA expressions of Nrf2 and HO-1 after stimulation with LPS (**Figure 6B**). Relative Nrf2 and HO-1 protein levels were also significantly increased in TIPE2-overexpressing macrophages (**Figure 6D**). In addition, TPE2 overexpression induced significantly increased protein levels of nuclear Nrf2 in M1 macrophages (**Figure 6E**). Immunocytochemistry analysis showed that M1 macrophages with TIPE2 overexpression showed more Nrf2 staining, especially nuclear Nrf2 staining (**Figure 6F**). However, silencing TIPE2 resulted in reduced expression levels of Nrf2, HO-1 and nuclear Nrf2 in LPS-induced M1 macrophages (**Figures 6A, C, E, F**). These data suggested that TIPE2 enhanced activation of the Nrf2/HO-1 pathway in M1 polarization.

### TIPE2 Inhibits M1 Macrophage–Related Inflammation by Enhancing Nrf2/HO-1 Activation

**Figure 7:**
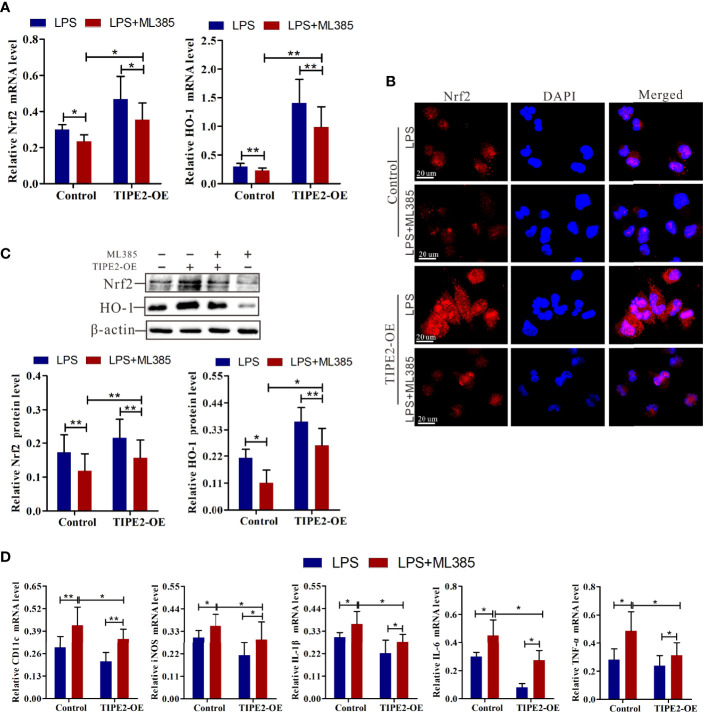
Nrf2 inhibitor weakened the anti-inflammatory role of TIPE2 in M1 macrophage polarization. **(A)** Expression of Nrf2 and HO-1 in M1 macrophages by RT-qPCR following treatment with the Nrf2 inhibitor ML385 (5 nmol) for 48 h. **(B)** Measurement of Nrf2 expression level in M1 macrophages by immunocytochemistry analysis following treatment with ML385 (5 nmol) for 48 h. **(C)** The protein levels of Nrf2 and HO-1 in M1 macrophages were detected by western blotting analysis following treatment with ML385 (5 nmol) for 48 h. **(D)** Measurement of CD11c, iNOS, IL-1β, IL-6, and TNF-α expression levels in M1 macrophages by RT-qPCR following treatment with ML385 (5 nmol) for 48 h. Statistics were performed on pooled data from at least three independent experiments. Error bars represent the standard deviations of the means. A comparison of two groups was performed using Student’s t-test. Comparison of the means among three groups was performed using one-way ANOVA followed by *post-hoc* test (LSD or Tamhane’s T2). ^*^P<0.05, ^**^P<0.01. TIPE2, tumor necrosis factor α–induced protein 8–like protein 2; Nrf2, nuclear factor erythroid 2–related 2; HO-1, heme oxygenase–1; RT-qPCR, reverse transcription–quantitative polymerase chain reaction; shRNA, short hairpin RNA; TIPE2-OE, TIPE2 overexpression; iNOS, inducible nitric oxide synthase; IL, interleukin; TNF-α, tumor necrosis factor–α; CTRL, control vector; ANOVA, analysis of variance; LSD, least significant difference.

ML385 is a specific Nrf2 inhibitor and inhibits activation of the Nrf2 pathway (32). To assess whether TIPE2 inhibits the M1 polarization of macrophages by targeting the Nrf2/HO-1 pathway, we treated macrophages with ML385 (5 nmol) for 48 h to block Nrf2 pathway activation. ML385 treatment decreased the up-regulated expression in HO-1 and Nrf2 mRNA induced by overexpression of TIPE2 in M1 macrophages (**Figure 7A**). ML385 treatment also reduced the increase in Nrf2, and HO-1 protein levels induced by overexpression of TIPE2 (**Figure 7C**). Moreover, M1 macrophages with ML385 treatment exhibited less Nrf2 staining compared with control M1 macrophages, but overexpressing TIPE2 weakened the decrease in Nrf2 expression induced by ML385 treatment in M1 macrophages (**Figure 7B**). Under M1 polarization conditions, TIPE2-overexpressed M1 macrophages had higher expression levels of Nrf2 and HO-1 after ML385 treatment (**Figures 5A, C**). ML385 treatment attenuated the down-regulated expression of M1 genes (CD11c and iNOS genes) induced by TIPE2 overexpression in macrophages following LPS stimulation (**Figure 7D**). Moreover, ML385 treatment significantly diminished the decrease in IL-1β, IL-6, and TNF-α mRNA levels induced by TIPE2 overexpression in M1 macrophages (**Figure 7D**). Upon treatment with ML385, TIPE2-overexpressing M1 macrophages had lower expression levels of CD11c, iNOS, IL-1β, IL-6, and TNF-α compared to control M1 macrophages (**Figure 7D**).

Together, these data indicated that blocking Nrf2 weakened the anti-inflammatory role of TIPE2 in M1 macrophages polarization. Therefore, these findings suggest that TIPE2 inhibits M1 macrophage differentiation and related inflammation by enhancing Nrf2/HO-1 pathway activation.

## Discussion

A previous study found that TIPE2 expression in PBMCs of asthmatic children was decreased (22). Another study, however, found that TIPE2 levels were higher in the polyps of patients with asthmatic Eos CRSwNP compared with non-asthmatic Eos CRSwNP (23). In contrast, our data showed that the TIPE2 level in sputum was comparable between asthma patients and healthy individuals. However, an inverse expression pattern of TIPE2 in sputum of NA and EA was observed. Interestingly, sputum TIPE2 levels in MA were significantly higher than those in NA but was not notably different from those in EA, which may be caused by the different ratio of sputum neutrophils and eosinophils. The heterogeneity of asthma airway inflammation or differences in asthma phenotypes could account for the disparities in TIPE2 levels in asthma patients. Significantly dissimilar infiltration of inflammatory cells and cytokines has been observed in different inflammatory phenotypes of asthma (34–36). TIPE2 in sputum of asthma patients positively correlated with pro-eosinophilic inflammation cytokines but negatively correlated with pro-neutrophilic inflammation cytokines. Abnormal expression of TIPE2 may play a regulator role in neutrophilic and eosinophilic inflammation in asthma.

Sputum TIPE2 level in EA patients was significantly higher compared with levels in PA patients. TIPE2 expression in sputum neutrophils, macrophages, and eosinophils in EA patients was higher than levels in NA patients. In sputum eosinophils of all phenotypes, TIPE2 was expressed at higher levels compared with that in sputum macrophages. Previous study showed that upregulated expression of TIPE2 in the polyps of Eos CRSwNP patients was positively related to eosinophil numbers, thereby aggravating local eosinophilic inflammation and disease severity (23). However, another study found that TIPE2 may enhance the immune-suppressive activity of Tregs, thereby extenuating eosinophil accumulation in the airway and reducing the severity of asthma (21). These studies exhibited a contradictory role of TIPE2 in eosinophilic airway inflammation in asthma (22, 23), which could be attributed to the heterogeneity of asthma inflammatory phenotypes. Thus, we assessed the correlation between sputum TIPE2 levels and cytokines that correlated with eosinophilic and neutrophilic inflammation in asthma. In this study, sputum TIPE2 levels were significantly increased in patients with EA and positively correlated with sputum IL-4, IL-5, and IL-13 that promoted eosinophilic inflammation in asthma (2). In addition, the sputum TIPE2 levels were positively correlated with sputum eosinophils, blood eosinophils and FeNO value, which prompts a positive correlation between TIPE2 with eosinophilic inflammation. However, TIPE2 was also positively correlated with anti-inflammatory cytokine IL-10. More researches needed to explore the role of TIPE2 in eosinophilic inflammation in asthma.

TIPE2 was negatively correlated with neutrophilic inflammation, which could be confirmed by the inhibitory role of TIPE2 on M1 inflammation. In this study, sputum TIPE2 levels were significantly decreased in NA patients and negatively correlated with sputum IL-1β and TNF-α. In sputum neutrophils of all phenotypes, TIPE2 was expressed at lower levels compared with that in sputum macrophages. Additionally, macrophages in NA patients showed lower TIPE2 expression compared with PA patients, while macrophages in EA patients had higher TIPE2 expression. The result was consistent with down-regulated expression of TIPE2 in M1 macrophages that can release neutrophilic inflammation factors and aggravate neutrophilic inflammation in asthma (3). TIPE2 was shown to inhibit macrophage apoptosis and M1 phenotype polarization *in vitro* by decreasing the levels of monocyte chemoattractant protein (MCP)-1, TNF-α, IL-6, IL-1β, and IL-12 (19, 37, 38). TIPE2 may inhibit neutrophilic inflammation in asthma.

Classical (M1) polarization of macrophages and related cytokines play a predominant role in airway inflammation in non-allergic asthma (11). Nrf2, a major transcription factor, promotes the expression of the cytoprotective gene HO-1, thereby inhibiting M1 macrophage polarization and reducing the expression of inflammatory cytokines (26). We found that TIPE2 enhanced activation of the Nrf2/HO-1 pathway in macrophages and down-regulated the expression of M1 genes and M1 inflammation cytokines stimulated by LPS. M1 polarization of macrophages and related cytokines play a predominant role in neutrophilic inflammation (38). *In vivo*, TIPE2 inhibited lipopolysaccharide-induced pulmonary cell apoptosis and neutrophil infiltration, thereby weakening lung inflammation and structure injury of mice (17). Exogenous TIPE2 treatment decreased the levels of serum IL-1β, IL-6 and TNF-α in acute lung injury mice, inhibiting lung inflammation (39). Furthermore, neutrophils were increased significantly in the cornea of *TIPE2^-/-^* keratitis mice after *Pseudomonas aeruginosa* infection (20). Additionally, Nrf2 pathway activation notably suppressed neutrophil recruitment to sensitized skin in hypersensitivity mice (40). More importantly, in the ovalbumin-induced asthma model, Nrf2 deficiency led to significantly increased levels of neutrophils and eosinophils in bronchoalveolar lavage fluid and lung tissues of mice, and aggravated oxidative stress, airway inflammation, and AHR in mice (41, 42). In short, TIPE2 inhibits these pro-inflammatory cytokines, which can promote neutrophils to the lung, exacerbating airway inflammation in NA (43). TIPE2 may target Nrf2/HO-1 pathway activation to inhibit M1 macrophages inflammation, thereby mitigating airway neutrophilic inflammation in asthma. Together, our results demonstrate an inhibitory role of TIPE2 on M1 inflammation through TIPE2 targeting Nrf2/HO-1 pathway activation and indicate that alterations in TIPE2 expression may lead to an interchange between the asthma inflammatory subtypes.

In conclusion, this study revealed that TIPE2 expression level was highly down-regulated in NA and was negatively correlated with inflammatory factors (IL-1β and TNF-α). *In vitro* analyses showed that TIPE2 impeded LPS-induced M1 macrophage differentiation and related inflammation by targeting activation of the Nrf2/HO-1 pathway. Aberrant expression of TIPE2 may target the Nrf2/HO-1 pathway to inhibit neutrophilic inflammation in asthma. Additional research will be required to elucidate the precise function of TIPE2 in each of these distinct asthma phenotypes.

## Data Availability Statement

The original contributions presented in the study are included in the article/supplementary material. Further inquiries can be directed to the corresponding author.

## Ethics Statement

The studies involving human participants were reviewed and approved by the Ethics Committee of the Second Hospital of Jilin University (approval number: 2016-34). The patients/participants provided their written informed consent to participate in this study.

## Author Contributions

BS drafted the manuscript. YH, WL, HD and MX reviewed and critically revised the manuscript for important intellectual content. PG provided substantial contributions to the study conception and design and confirmed final approval of the version to be published. All authors contributed to the article and approved the submitted version.

## Funding

This research was funded by the Natural Science Foundation of Jilin Province (20210101460JC), National Natural Science Foundation of China (82070037), Jilin Province Natural Science Foundation (202000201384JC), Jilin Province Development and Reform Commission Plan (2019C047-7), and Jilin Provincial Department of Finance, Provincial Talent Project (2019SCZT033). The design of the study and writing of the manuscript were performed in accordance with the rules of the funding bodies.

## Conflict of Interest

The authors declare that the research was conducted in the absence of any commercial or financial relationships that could be construed as a potential conflict of interest.

## Publisher’s Note

All claims expressed in this article are solely those of the authors and do not necessarily represent those of their affiliated organizations, or those of the publisher, the editors and the reviewers. Any product that may be evaluated in this article, or claim that may be made by its manufacturer, is not guaranteed or endorsed by the publisher.

## Abbreviations

EA, eosinophilic asthma; NA, neutrophilic asthma; MA, mixed granulocytic asthma; PA, paucigranulocytic asthma; Th, T helper cell; IL, interleukin; IgE, immunoglobulin E; GINA, Global Initiative for Asthma; TNF-α, tumor necrosis factor-α; TNFAIP8, tumor necrosis factor-alpha-induced protein-8; TIPE, TNFAIP8-like; PBMC, peripheral blood mononuclear cell; CRSwNP, chronic rhinosinusitis with nasal polyps; Eos, eosinophilic; Nrf2, nuclear factor E2-related factor 2; HO-1, heme oxygenase-1; LPS, lipopolysaccharide; FeNO, fractional exhaled nitric oxide; IFN, interferon; PMA, phorbol-12-myristate-13-acetate; shRNA, small hairpin RNA; ICS, inhaled corticosteroid; iNOS, inducible nitric oxide synthase; MCP-1, monocyte chemoattractant protein 1.
